# Removing Pollutants from Sewage Waters with Ground Apricot Kernel Shell Material

**DOI:** 10.3390/ma15103428

**Published:** 2022-05-10

**Authors:** Ildar Shaikhiev, Karina Shaykhieva, Svetlana Sverguzova, Ekaterina Fomina, Yuriy Vinogradenko, Roman Fediuk, Mugahed Amran, Alexander P. Svintsov, Afonso Rangel Garcez de Azevedo, Murali Gunasekaran

**Affiliations:** 1 Department of Environmental Engineering, Kazan National Research Technological University, 420074 Kazan, Russia; ildars@inbox.ru (I.S.); shaknv1978@mail.ru (K.S.); 2 Department of Industrial Ecology, Belgorod State Technological University, V.G. Shukhov, 308012 Belgorod, Russia; pe@intbel.ru (S.S.); natmir16@yandex.ru (Y.V.); 3 Polytechnic Institute, Far Eastern Federal University, 690922 Vladivostok, Russia; 4 Peter the Great St. Petersburg Polytechnic University, 195251 St. Petersburg, Russia; murali_220984@yahoo.co.in; 5 Department of Civil Engineering, College of Engineering, Prince Sattam Bin Abdulaziz University, Alkharj 16273, Saudi Arabia; m.amran@psau.edu.sa; 6 Department of Civil Engineering, Faculty of Engineering and IT, Amran University, Amran 9677, Yemen; 7 Department of Civil Engineering, Peoples’ Friendship University of Russia (RUDN University), 117198 Moscow, Russia; svintsovap@rambler.ru; 8 LECIV—Civil Engineering Laboratory, UENF—State University of the Northern Rio de Janeiro, Av. Alberto Lamego, 2000, Campos dos Goytacazes, Rio de Janeiro 28013-602, Brazil; afonso.garcez91@gmail.com

**Keywords:** apricot kernel shell, metal ion, dye, adsorption, modification

## Abstract

For the first time, a comprehensive review of the literature data on the use of apricot (*Prunus armeniaca*) biomass components as a sorption material for the treatment of wastewater and environmental water from various pollutants is carried out in the present study. In addition to a comprehensive analysis of contemporary studies, the current work carried out its own microstructural and energy dispersive studies. It shows that apricot kernel shell is a promising raw material for obtaining sorption materials that can be used to extract various pollutants from aqueous media. The parameters of sorption interaction are presented, at which the highest rate of removal of pollutants was achieved. It is shown that the sorption capacity of apricot biomass components can be increased by modifying it with various chemical reagents, as well as other physical and physicochemical methods. We reveal that most publications consider the use of the latter as a raw material for the production of activated carbons. It is established that the surface area and total pore space of activated carbons from apricot kernel shells depend on the modes of carbonization and activation. It is shown that activated carbons are effective adsorbents for removing various pollutants (metal ions, dyes, oil and oil products) from aqueous media. It was found that the adsorption isotherms of pollutants in most cases are best described by the Langmuir and Freundlich models, and the process kinetics is most often described by the pseudo-second-order model. The possibility of improving the sorption characteristics of apricot biomass during chemical or physicochemical treatment is also shown.

## 1. Introduction

Nowadays, various technologies in the field of environmental protection are rapidly developing in the world community. One of the innovative trends is the use of industrial and agricultural waste as reagents for the removal of pollutants from gaseous and aqueous media. [1,2].

Of special interest are wood biomass components, such leaves [1,2,3], conifers’ needles [4,5,6] and cones [7,8,9] and fruit peels [10,11,12]; and wood processing waste, such as sawdust [13,14,15], fruit kernels left after fruit processing [16,17,18] and nut shells [19,20,21], which are generated annually in large quantities, are cheap and have a renewable raw-material base.

**Figure 1:**
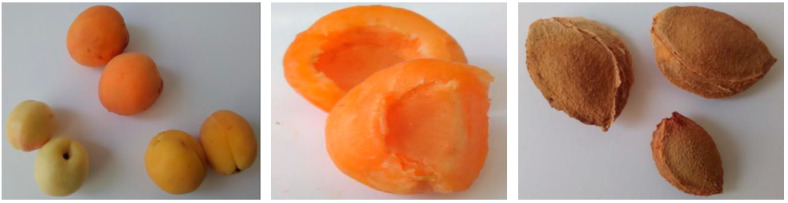
Apricot fruits and kernel shells.

A typical deciduous tree, widely occurring in the southern areas of the Russian Federation, as well as in Central Asian and Caucasian countries, is common apricot (*Prúnus armeníaca*), a fruit tree of the *Armeniaca* section, *Prunus* genus, *Rosaceae* family. The fruits are juicy, yellow to red («apricot-colored») drupes, roundish, elliptical or obovoid, with a lengthwise groove. The kernel is thick-walled, smooth or grainy (Figure 1).

The apricot tree has a long lifespan, up to 100 years in a warm climate; the fruiting begins at the age of 3–5 years and last for up to 30–40 years. The trees are drought-resistant (due to their deep roots), so they can grow in a hot climate with scarce rainfall [22].

Despite the dozens of articles found on the potential of using apricot kernels as an absorber of various contaminants, it seems necessary to review the effectiveness of various methods of processing this material. However, the adsorption of iron ions by the leaves of deciduous trees, including apricot, has been studied. It was determined that, at the initial concentration of Fe^3+^ ions of 100 mg/dm^3^, the highest adsorption capacity of *Prunus armeniaca* leaves for the mentioned ions corresponded to 39.8 mg/g [23]. The adsorption of Cu^2+^ ions with apricot tree sawdust was also studied. It was found that the adsorption capacity of *Prunus armeniaca* sawdust for copper ions was relatively low (~4.5 mg/g), and the adsorption isotherm was the most accurately described with the Langmuir model [24].

**Figure 2:**
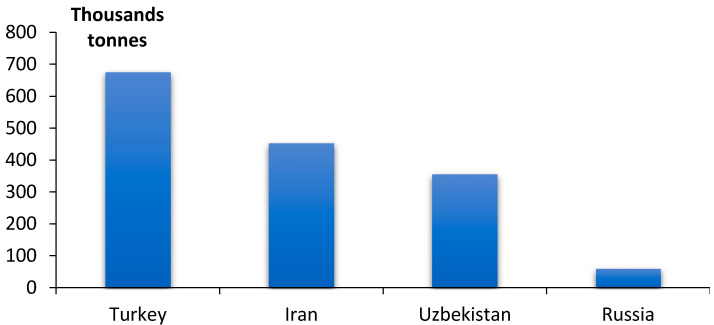
Apricot production in some countries (2018).

Some more publications are devoted to using apricot kernel as a sorption material. It has been noted that the world production of apricots in 2018 made up over 2 mln tons [25], with over 676 thousand tons being produced in Turkey, over 453 thousand tons in Iran and over 356 thousand tons in Uzbekistan (Figure 2). In Russia, the production of apricot fruits is somewhat over 60 thousand tons [25].

Naturally, by processing thousands of tons of apricots, thousands of tons of kernels are generated as a waste product. In this work, the ground native apricot kernel shells were researched for removing heavy metal ions and dyes from water media.

Meanwhile, apricot fruit shells containing cellulose can have good sorption properties with respect to various pollutants. Therefore, the study of the possibility of using apricot kernel shell material for the purification of polluted waters is an urgent task.

The goal of this work is to study the possibility of using native or modified apricot kernel shell material in water treatment technologies. To achieve this goal, it is necessary to solve the following tasks:

- Analyze the ways to increase the sorption capacity of apricot kernel shell biomass;
- Perform own microstructural and energy dispersive studies;
- Improve the sorption properties of apricot kernel biomass;
- Use the activated biomass of apricot kernel shells to remove heavy metal ions from aquatic environments;
- Extract dyes from the aqueous media by the biomass of apricot the kernels;
- Purify the aqueous media from pharmaceutical products and pesticides;
- Extract the oil and oil products.

## 2. Materials and Methods

In addition to a comprehensive analysis of the literature sources, the finely dispersed (<0.08 mm) powder of apricot kernel shell biomass was used in the current work. Microphotographs of the surface were obtained using a Hitachi-8-800 instrument (Hitachi, Tokyo, Japan) combined with a personal computer, and high-resolution scanning electron microscope MIRA 3 LMU (Tescan, Brno, Czech).

Energy-dispersive studies were carried out using a Quanta200-3D scanning electron microscope (FEI Company, Hillsboro, OR, USA) with an energy-dispersive determination of the chemical composition of the materials.

## 3. Results and Discussion

### 3.1. Ways to Increase the Sorption Capacity of the Apricot Kernel Shell Biomass

**Figure 3:**
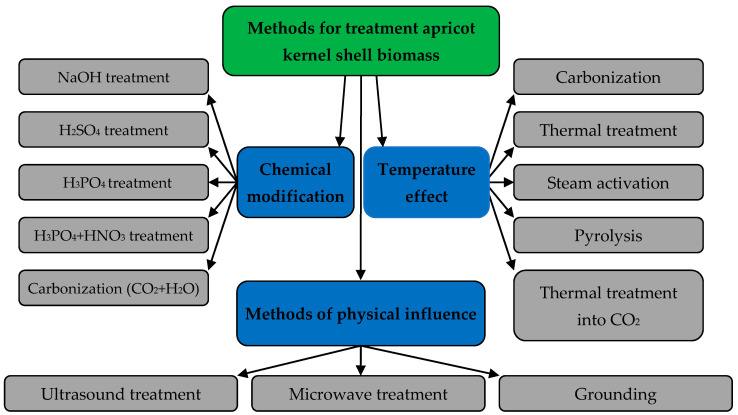
Methods used for the treatment of apricot kernel shell biomass.

The native biomass of crushed apricot kernels can be used to extract various pollutants from aquatic environments, but its sorption capacity is low. Therefore, to improve the sorption properties of biomass, it is possible to apply various methods of its processing, among which thermal, physical and chemical methods of processing are common (Figure 3) [22,23,24,25,26,27,28].

The following methods of treatment of the biomass of apricot kernel shells were studied in detail in the work:

- Thermal treatment at 250 °C;
- Ultrasonic treatment of 200 W during 15 min with a frequency of 22 kHz;
- Alkali treatment with NaOH solution on the sorption capacity of apricot kernel shells for Cu^2+^, Pb^2+^ and Zn^2+^ ions;
- Treating the sorption material sample with microwave radiation of 300 W for 10 min.

### 3.2. Own Microstructural Studies of Some Physicochemical Properties of the Apricot Kernel Shell Biomass

**Figure 4:**
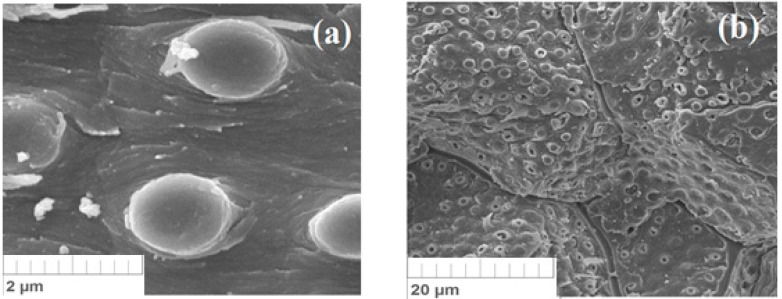

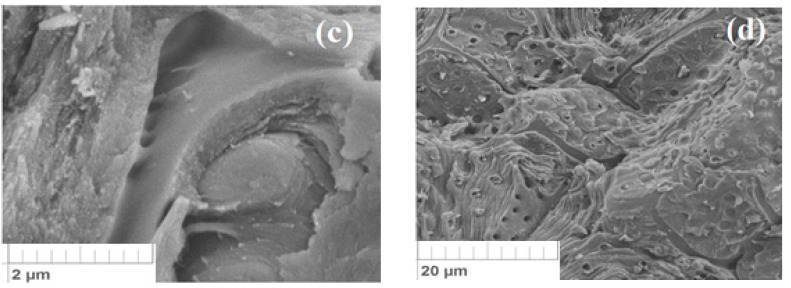
SEM analysis of the microstructure of surfaces of original (**a**,**b**) and burnt (**c**,**d**) apricot kernel shell biomass.

SEM images (Figure 4) of different areas of the surface of the crushed biomass were studied: original (a, b) and fired at 250 °C in air (c, d). It was revealed that both the initial and heat-treated biomass have a rough surface, on which fibers, pores of various shapes and sizes, and the presence of space between the fibers are preserved.

The specific structure of the biomaterial makes it possible to retain molecules and ions of pollutants. At the same time, heat treatment of the material leads to an increase in the defectiveness of the surface (Figure 4c,d), increasing the sorption properties for many substances.

**Figure 5:**
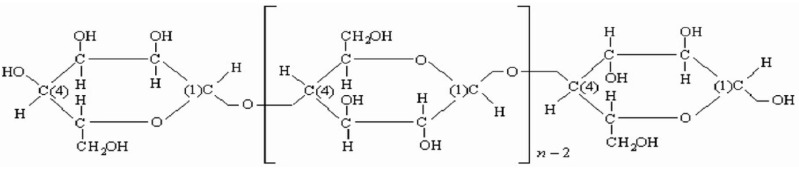
Chemical structure of cellulose molecule formula.

The structure of the biomass of apricot kernel shells contains a large proportion of cellulose, the original polymer, the elementary units of which have the following chemical formula (-C_6_H_10_O_5_-)_n_. The chemical structure of the formula of the cellulose molecule is shown in Figure 5.

A characteristic feature of cellulose is the presence of three hydroxyl groups in each elementary unit –OH^−^. The functional hydroxyl group is able to interact with heavy metal ions due to the replacement of hydrogen cations by metal ones according to the following scheme:

zR·OH + Me^z+^ + zOH^−^ → zR·OMe + zH_2_O

**Figure 6:**
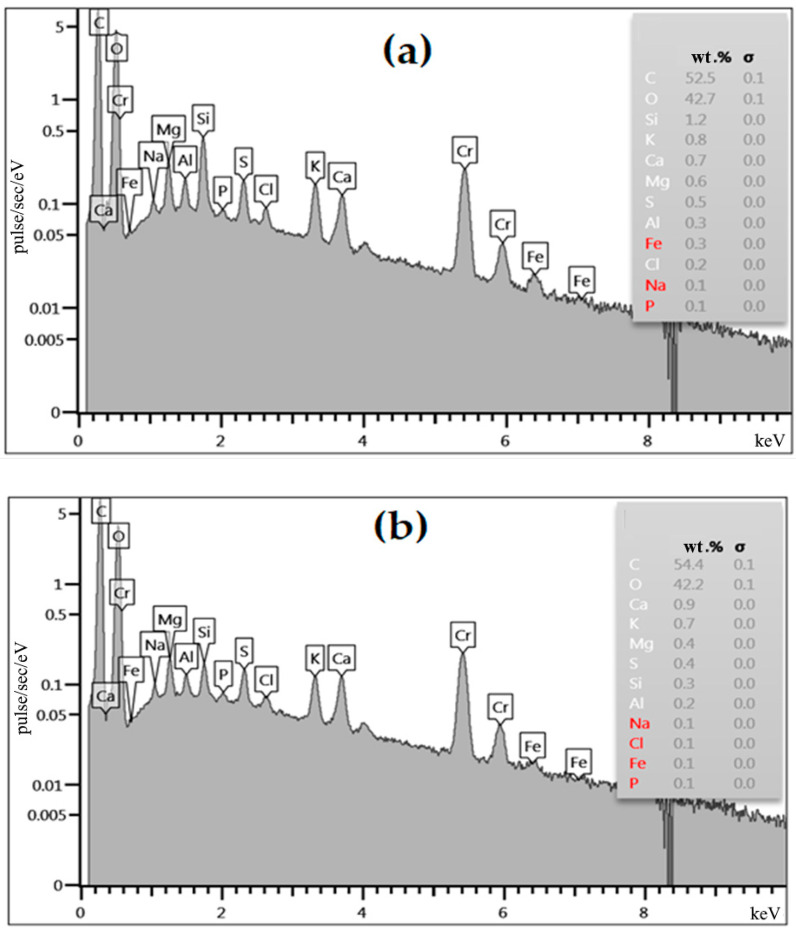
Energy-dispersive spectrum of the original (**a**) and fired biomass of apricot kernel shells at 250 °C (**b**).

Consequently, wastewater treatment using the obtained material occurs not only as a result of physical sorption, but also chemisorption processes. Figure 6 shows the energy-dispersive spectra of the original (a) and fired biomass of apricot kernels at 250 °C. According to the obtained data, the biomaterial of both samples contains chemical elements, such as carbon, oxygen and (in small quantities) silicon, potassium, calcium, magnesium, sulfur, aluminum, iron, chlorine, sodium and phosphorus. Moreover, the chemical compositions of both samples slightly differ from each other.

### 3.3. Improving the Sorption Properties of the Apricot Kernel Shell Biomass

The adsorption of Cr^6+^ ions with ground native shells of *Prunus armeniaca* kernels was studied. It was determined that, at the initial concentration of 5 mg/dm^3^, pH = 2, 30 min of contact time and adsorbent dosage of 20 g/dm^3^, the maximum sorption capacity amounted to 0.037 mg/g. The adsorption isotherms were the most accurately described with the Freundlich model (R^2^ = 0.990), and the process kinetics fitted the pseudo-second-order model [26].

The sorption characteristics of ground apricot kernels shells can be increased by ultrasonic treatment. Thus, the ultrasonic treatment of 200 W during 15 min with a frequency of 22 kHz helps to increase the maximum sorption capacity from 6.6 mg/g to 9.9 mg/g for Cr^6+^ ions. Similar to the previous work, it was found that the maximum sorption capacity was observed at pH = 2, at the initial concentration of Cr^6+^ ions of 75 mg/dm^3^. It was determined that, at the initial concentrations of these ions of 25, 50 and 75 mg/dm^3^, the degrees of their removal from the native sorption material were 45.6%, 32.4% and 25.8%, and 62.4%, 47.2% and 38.4% after ultrasonic treatment, respectively [27].

The native apricot kernels shells were also studied as a sorption material for removing Cu^2+^ ions in static conditions. It was found that the maximum sorption capacity, equal to 4.5 mg/g, was achieved at pH = 5, the initial concentration of Cu^2+^ ions of 60 mg/dm^3^ and sorption material dosage of 6 g/dm^3^. It was determined that the main process of this mechanism was ion exchange. The adsorption isotherm was well described with the Langmuir model, and the sorption kinetics fit the pseudo-second-order model [28]. At the initial concentration of copper ions of 120 mg/dm^3^ and an apricot kernel shell biomass dosage of 0.5 g/dm^3^, the removal degree of metal ions made up over 42% [29]. By processing the experimental data by means of the artificial neural network (ANN) method, it was determined that the maximum sorption capacity of ground apricot kernel shells for Cu^2+^ ions amounted to 5.9 mg/g, and the removal efficiency made up 26.9% at the adsorbent dosage of 0.3 g/dm^3^ [30].

The sorption capacity of the adsorbent for Cu^2+^ ions can be increased by the chemical modification of apricot kernel shell biomass. Thus, it is noted that treating the latter with NaOH helps to increase the maximum sorption capacity by more than 4 times, from 2.5 mg/g to 10.8 mg/g [31].

The removal of Pb^2+^ ions with apricot kernel shells and other sorption materials made of agricultural raw material processing waste was considered. It was determined that the maximum adsorption capacity of apricot kernel shells for lead ions made up 22.78 mg/g [32].

**Figure 7:**
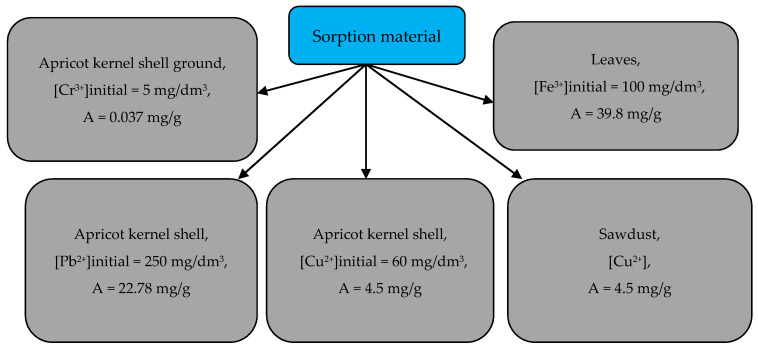
Sorption capacity of leaves, sawdust and apricot kernel shells in relation to some heavy metal ions.

The efficiency of removing Cu^2+^ and Pb^2+^ ions with apricot kernel shells under the same conditions (the initial concentration of metal ions was between 50 to 250 mg/dm^3^, adsorbent dosage was 20 g/dm^3^ and pH was 2–6) was studied. In each case of removing the metal ions, the following relation was observed: Pb^2+^ > Cu^2+^ [33]. Data on the sorption capacity of the biomass of leaves, sawdust and kernel shells of apricots are shown in Figure 7 [32,33,34,35].

**Figure 8:**
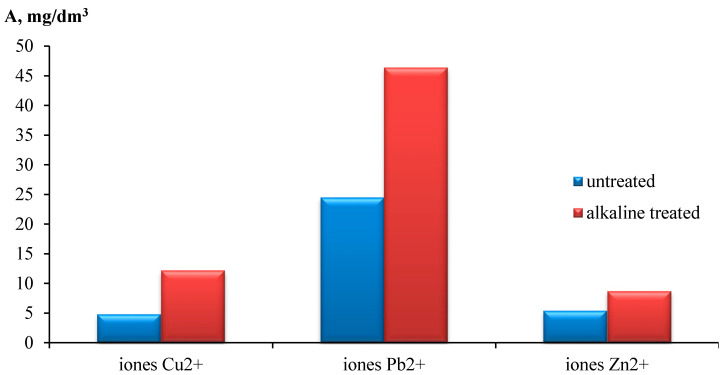
Influence of alkaline treatment of apricot kernel shells on the sorption capacity with respect to ions Cu^2+^, Pb^2+^ and Zn^2+^.

The influence of alkali treatment with an NaOH solution on the sorption capacity of apricot kernel shells for Cu^2+^, Pb^2+^ and Zn^2+^ ions was studied. It was determined that the alkali treatment increased the maximum sorption capacity from 4.83, 24.53 and 5.42 mg/g to 12.25, 46.45 and 8.73 mg/g, respectively (Figure 8). The chemical analysis demonstrated that the alkali treatment the caused decomposition of hemicellulose (its content was reduced from 19.2 to 3.5%), and increased the surface area and the porosity of the sorption material. It was supposed that the main mechanisms of this are ion exchange and complex formation [34].

The adsorption of Methylene blue dye with ground apricot kernels, treated with an alkali solution or microwave radiation, was carried out. It was determined that the highest degree of the dye’s removal was observed with the sorption material sample treated with an NaOH solution at the ratio of 3:1. At the initial concentration of the dye, 150 mg/dm^3^, the sorption capacity made up ~70 mg/g, and the removal degree ~48%. The sorption characteristics were improved by treating the sorption material sample with microwave radiation of 300 W for 10 min. The adsorption capacity after this treatment was 95 mg/g, and the removal degree of the dye was over 65%. It was determined that, at the adsorption temperatures of 25–55 °C, adsorption isotherms were the most accurately described with the Langmuir model and, at the initial concentrations of 100–250 mg/dm^3^, the process kinetics was described with the pseudo-second-order model [35].

One of the ways of using agricultural processing waste is by obtaining activated carbons or activated charcoals (ACs) from them [36,37,38,39,40,41,42]. The kernel shells of *Prunus armeniaca* were also used for obtaining activated charcoals or thermally modified sorption materials for removing pollutants from sewage waters. The selection of this material was explained by the fact that an apricot kernel shell is a low-ash material, and its high true density determines the possibility of obtaining solid adsorbents on its basis. Additionally, this kind of raw material is characterized with already having a natural system of pores and channels in its structure, which can be developed with various methods of carbonization and subsequent activation.

For activated charcoal production, usually either a single-stage process of raw hydrocarbon carbonization or a two-stage process, with the subsequent activation of carbonizate, is applied. Two AC samples of apricot kernels were prepared by means of carbonization with the subsequent vapor activation or by means of single-stage pyrolysis/activation in steam. It was determined that the two-stage method allowed us to obtain ACs with a larger amount of mesopores and macropores and a larger surface area [43,44].

The activated charcoal was obtained as a result of the carbonization apricot kernel shells at 700 °C for 2 h and thermal activation in a CO_2_ and H_2_O atmosphere (85:15) at a temperature of 800 °C for 1 h. The output of the activated coal was 35%. It was determined that the pore volume of the obtained activated coal was 1 cm^3^/g [45].

The carbonization of the apricot kernels was carried out at a temperature of 550 °C in vacuum, and the subsequent activation with water vapor was performed at 850 °C for 1 h. It was determined that the surface area of the obtained ACs made up 506 m^2^/g, a total pore volume of 0.305 cm^3^/g, a micropore volume of 0.226 cm^3^/g and the average size of the micropores was 1.321 nm [46].

Ground apricot and peach kernels with particle sizes of 0.3–3.0 mm were used for obtaining activated carbon. Carbonization was performed at temperatures of 500–950 °C for 1–3 h, and activation at 800–850 °C for 1.0–5.0 h, using water vapor and a gas–vapor mixture. It was experimentally determined that the optimum conditions for the carbonizate activation of the apricot kernels was the temperature of 850 °C for 3 h. It was found that the obtained activated carbons presented adsorption activity for the Methylene blue dye (6.39 m^2^/g) and iodine (25.34%) [47].

To obtain activated coal, the ground fruit kernel shells, including *Prunus armeniaca* kernel shells, with moisture content of 15–20% and particle sizes of 1.0–1.5 mm, were treated in the reactor at temperatures of 350–400 °C and pressures of 15–22 MPa, and then the pyrolyzed mass was treated with the gas–vapor mixture. It was noted that the obtained activated carbons had an increased chemical stability, which allows them to be revivified many times, and the mechanical strength increased by 15–20% with a high porosity of 0.6–1.0 cm^3^/g [48].

Unlike the previous work, the present study studied the carbonization processes in vacuum and the gas–vapor activation of apricot kernel shells. The study of the linear adsorption/desorption isotherms in the samples, obtained by the gas–vapor activation of apricot kernel carbonizates, shows that the total specific surface by the BET method made up 560 m^2^/g, a total pore volume of 0.3 cm^3^/g and a micropore volume (calculated by the Dubinin–Radushkevich method) of 0.266 cm^3^/g [49].

The carbonization of apricot kernels was carried out at a temperature of 500 °C for 1 h, and the obtained carbonizate was activated by using water vapor and a gas–vapor mixture at 950 °C for 1.5 h. It was determined that the specific surface of the AC made up 1390 m^2^/g, the adsorption activity for Methylene blue dye was 356 mg/g and iodine was 1083 mg/g, which exceeds the characteristics of commercial-grade activated carbons, such as OU-A, BAU-A and Karbolen [50].

Apart from the two-stage method of obtaining AC from lignocellulosic raw material, a three-stage method of producing activated coal for medical use from apricot kernels was suggested. The necessity of carrying out the carbonization process in two steps is demonstrated: the low-temperature stage was performed at 350–400 °C and the high-temperature stage at 800–850 °C, with the subsequent gas–vapor activation of the carbonized product with water vapor. During the first stage of the carbonization process, the main mass of the volatile substances was removed, and the formation of the primary porous structure of the carbon material occurred at the high-temperature stage. The findings of researching the synthesized carbon materials confirmed the formation of a uniform microporous structure of the carbon materials, obtained from fruit kernels [51].

To increase the surface area of the AC, it is suggested that the carbonizate obtained from the agricultural raw material processing waste, including the apricot kernels, be activated by microwave radiation. It was found out that the AC made of apricot kernels has the specific surface area, determined by the BET method, of 529 m^2^/g, and the total pore and micropore volumes were 0.26 and 0.19 cm^3^/g, respectively [52]. The application of microwave radiation as an activation method considerably reduces energy consumption and activation time in comparison with conventional methods. In fact, the optimum power of microwave radiation varies from 400 to 650 W, and the radiation time from 3 to 6 min.

Nevertheless, it is noted that the two-stage and three-stage methods used for obtaining activated carbons from solid raw materials are cost-inefficient. In this regard, the research in obtaining ACs from *Prunus armeniaca* kernels shells with a single-stage method was performed.

The carbonization of agricultural waste, including apricot kernel shells, was conducted in a nitrogen atmosphere at 550 °C. It was found that the carbonizate output made up 35.5%, the surface area, determined by low-temperature nitrogen adsorption with the BET method, was 17.1 m^2^/g and the average micropore size was 1.12 nm. It was determined that the maximum adsorption capacity for Methylene blue dye amounts was 10.8 mg/g and 48.0 mg/g for iodine [53]. The carbonization of apricot kernels at 850 °C for 1 h in a nitrogen atmosphere allowed us to obtain AC with a surface area of 328.6 m^2^/g, and a micropore volume and size of 0.15 cm^3^/g and 1.62 nm, respectively [54]. It was found that by increasing the treatment temperature of apricot kernels, the product output reduced, and the percentage content of the carbon and density of carbonizate increased [55].

**Figure 9:**
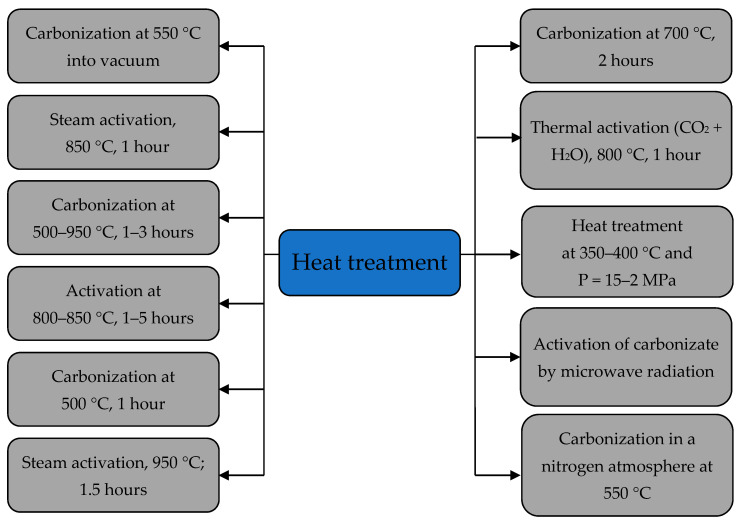
Some methods of heat treatment of apricot kernel shells.

The influence of sulfur content (0.024–0.04%) in various samples of the apricot kernels treated with a single-stage process of steam pyrolysis and activation at temperatures of 650–850 °C for 1–4 h was studied. It was determined that the AC with the largest surface area (1092 m^2^/g) was obtained from apricot kernel samples with a particle size of 1–3.35 mm and a sulfur content of 0.04% in conditions of activation at 800 °C for 4 h [56]. Figure 9 shows some ways to heat treat apricot kernel shells to increase their sorption capacity.

The research in developing a method of obtaining pelletized, spherical, activated coals based on agricultural waste, including apricot kernels, was conducted. The sorbent pelletizing process was performed by means of liquid dispergating of the composition, containing plant waste and a binder, for which a novolac phenol formaldehyde resin with a mass ratio of 1:5 was used, and then the obtained composition was pulverized into a sulfuric acid solution with a concentration of 30–35% for pellet curing. The latter was kept in the acid solution for 24–30 h, then the spherical pellets were separated from the liquid, washed with distilled water up to pH = 5–6 and air-dried at first, and then heat-treated at high temperatures. It was determined that the total pore volume made up 1.5 cm^3^/g, with the volumes of micro-, meso- and macropores of 0.430, 0.265 and 0.824 cm^3^/g [57].

### 3.4. Removal of Heavy Metal Ions from Aqueous Media by Activated Biomass of Apricot Kernel Shells

The activated carbons, obtained by the heat treatment of apricot kernels, were researched for removing metal ions from model and sewage waters.

The activated carbon, obtained from *Prunus armeniaca* kernels and having surface areas ranging from 900 to 1387 m^2^/g, was used for removing Au^+^ ions. The plotted adsorption isotherms with the initial concentrations of Au(I) ions from 20 to 150 mg/dm^3^ were the most accurately described with the Freundlich model. The values of the maximum sorption capacity of the AC for Au(I) ions at temperatures of 25–60 °C were calculated by the Langmuir equation and made up of 6.0 to 30.2 mg/g, respectively. The thermodynamic parameters of the process were determined: ΔGo = 0.048–0.134 kJ/mol, ΔHo = −85.71 kJ/mol and ΔSo = (−0288)–(−0.257) J/mol∙K. By using the experimental design method, the process conditions, at which the highest degree of gold ion removal was achieved, were determined: pH = 10.5, AC dosage—20 g/dm^3^ and adsorption time—3 h [58,59].

The adsorption process of Co^2+^ ions with the AC, obtained from apricot kernels, in static conditions at the initial ion concentrations of 10–80 mg/dm^3^, pH = 2–13.5, adsorbent dosage 5–50 g/dm^3^ and temperature 298–323 K, was studied. The maximum adsorption capacity for Co^2+^ ions, calculated by the Langmuir equation, made up 111.11 mg/g at pH = 9. It was determined that the adsorption isotherms were the most accurately described with the Langmuir model (R^2^ = 0.9993), and the process kinetics fit the pseudo-second-order model [60].

The removal of Cr^6+^ ions from model solutions with the initial concentrations of 10–40 mg/dm^3^ by means of fruit kernels-activated coals in dynamic conditions was researched. Depending on the solution’s flow rate through the sorbent layer, its thickness, and the initial concentration of chromium ions, the maximum sorption capacity varied from 3.15 to 10.64 mg/g. It was determined that the adsorption process was the most accurately described with the Thomas model [61].

The parameters, at which there are the highest sorption characteristics for removing Cr6+ ions in static conditions with activated carbon, made of apricot kernel shells, were determined. It was found that, at the initial concentration of Cr(VI) ions of 5 mg/dm^3^, the lowest final value ~2.75 mg/dm^3^ was achieved at pH = 7, the contact time was 30 min, the sorption material dosage was 0.25 g/dm^3^ and the temperature was 30 °C [62]. Activated carbons made of apricot kernel shells were studied for removing Cu^2+^ ions from model solutions with concentrations of 25–1000 ppm. It was determined that, at pH = 2–5 and temperatures of 290 and 308 K, the maximum sorption capacity values, calculated from the Langmuir equation, made up 23.64–48.01 mg/g [63]. The AC samples, obtained from apricot kernel shells, were studied for adsorbing Mn^2+^ ions. It was determined that, at a dosage of 2.5 g/dm^3^, the maximum sorption capacity value, calculated from the Langmuir equation, made up 10.2 mg/g at pH = 5–6 [64].

**Figure 10:**
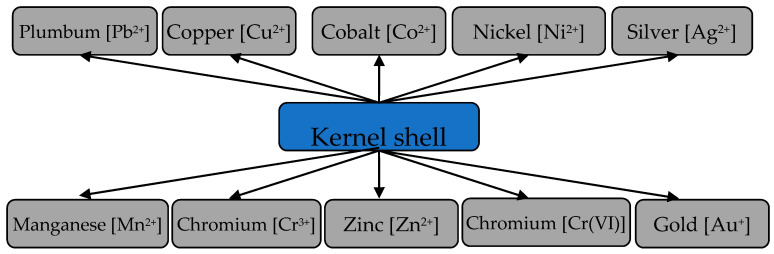
Some metals extracted from aquatic environments with activated carbon from apricot kernel shells.

Activated carbons, obtained from the apricot kernel shells, were also used for extracting Pb^2+^ ions [65,66,67]. It was determined that the AC, activated with sulfuric acid treatment, had a surface area of 393.2 m^2^/g, pore volume of 0.192 m^3^/g and sorption capacity for iodine and Methylene blue dye of 134 and 91 mg/g, respectively [65]. The sorption capacity amounted to 21.38 mg/g Pb^2+^ at pH = 6 [66]. It was found that the adsorption isotherm was the most accurately described with the Langmuir model, and the process kinetics fit the pseudo-second-order model [67]. It was determined by the thermodynamic characteristics that the adsorption process was endothermic and spontaneous. Figure 10 shows some metals extracted from the aqueous media with activated carbon from the apricot shells.

There are also some research data concerning the removal of thallium ions from model solutions with activated carbon, modified with Rhodamine B dye. It was found that this process occurred rapidly, and the equilibrium was established after 30 min. It was determined that the process kinetics corresponded to the pseudo-second-order model [68].

As follows from the above-mentioned data, the adsorption characteristics of the AC for various metal ions were vastly different. This is explained by the fact that activated carbons were obtained by various methods, have different sorption characteristics and the experiments were conducted under different conditions. It is much more informative to compare the adsorption characteristics when experiments are conducted under comparable conditions. In this regard, the data concerning the sorption capacity of two and more metals ions, obtained by using apricot kernel shells under the same experimental conditions, are presented below.

The research in obtaining and the application of AC made of fruit kernels, including apricot kernel shells, for extracting Ag and Au ions from cyanide-containing solutions, was conducted. It was experimentally deduced that the optimum conditions for fruit kernel raw material carbonization were T = 750–800 °C, and for activation were T = 850–860 °C. Activated coals have uniform microporous structure, and high values of characteristic energy absorption E^0^ = 26 kJ/mol at a micropore volume of W_0_ = 0.32 cm^3^/g. It was determined that the maximum sorption capacity for Ag ions made up 4.6 mg/g, and for Au ions 11.9 mg/g. It was found out that, in selection of gold and silver ions, the AC was as good as the AM-2B anionite (AM-2B anionite is a macroporous ion-exchange resin based on a copolymer of styrene with divinylbenzene, containing strong and weakly basic functional groups in its structure). Additionally, it was determined that the amount of the Ag and Au ions, absorbed with the activated carbon, considerably exceeded the adsorption of impurity metals ions (Co, Cu, Fe, Ni and Zn). The selectivity coefficient was K_sel_ = 90.16% [69]. The results of the integrated experimental data processing by the equilibrium-kinetic analysis method indicate the internal-diffusion nature of gold and silver ion adsorption by AC from the solutions [70].

Activated carbons made of apricot kernel shells were studied for extracting Al^3+^ and Zn^2+^ ions from model solutions. It was found that the maximum adsorption capacity is achieved at pH = 6–6.5. At the initial concentrations of Al^3+^ and Zn^2+^ ions of 200 and 300 mg/dm^3^, the maximum sorption capacities made up ~85 and 125 mg/g, respectively, and the extraction degree of these ions at the AC dosage of 2 g/dm^3^—over 95% in both cases. It was determined that the adsorption isotherms were the most accurately described with the Langmuir model, and the process kinetics described with the pseudo-second-order model. The calculated thermodynamic characteristics indicated the running of a spontaneous and endothermic process [71].

The adsorption of Cr^3+^ and Pb^2+^ ions with AC made of *Prunus armeniana* kernel shells, at varying process parameters, was studied. It was determined that the maximum sorption capacity, calculated from the Langmuir equation, made up for Cr^3+^ ions of 12.69 mg/g, and Pb^2+^ ions of 23.89 mg/g (the initial concentration of heavy metals ions was 50 mg/dm^3^, pH = 6, contact time—30 min/140 rpm, AC dosage was 4 g/dm^3^ and T = 22 °C). Adsorption isotherms are more accurately described with the Freundlich model; the process kinetics corresponds to the pseudo-second-order model [72].

The AC, activated with phosphoric acid, was used for the extraction of Cd^2+^, Ni^2+^ and Pb^2+^ ions from the model solutions. It was determined that the obtained activated coal had a total surface area of 1098 m^2^/g, and the total pore volume was 0.505 cm^3^/g. At the initial concentrations of Cd^2+^, Ni^2+^ and Pb^2+^ ions of 100 mg/dm^3^, the removal efficiency of the two first ions amounted to over 95%, nickel ions—50% at pH = 6, adsorption time of 30 min/140 rpm and AC dosage of 2 g/dm^3^. It was determined that the maximum sorption capacities, obtained during the experiments, were 45.83, 24.28 and 48.44 mg/g. Similar to the previous case, the adsorption isotherms are well described with the Freundlich model, and the process kinetics described with the pseudo-second-order model [73].

The adsorption of Cd^2+^, Cu^2+^, Pb^2+^ and Zn^2+^ ions from model solutions with the initial concentrations of the latter (0.4 mmol/dm^3^) individually and in mixtures was studied. As a result of the experiments that were conducted, it was determined that, for the individual metal ions adsorption, the values of the sorption capacity for the above-mentioned metal ions were arranged in a series: Cd^2+^ (0.042 mmol/g) > Cu^2+^ (0.041 mmol/g) > Pb^2+^ (0.039 mmol/g) > Zn^2+^ (0.038 mmol/g). In the case where heavy metals ions were adsorbed from a mixture with the initial concentration of each ion of 0.4 mmol/dm^3^, the sorption capacity values series were arranged in somewhat different sequence: Cu^2+^ (0.033) > Pb^2+^ (0.029) > Zn^2+^ (0.0061) > Cd^2+^ (0.0049) mol/g. Depending on the nature of the heavy metals ions, the adsorption isotherms of these ions are described with Langmuir, Freundlich and SIPS models [74]. The adsorption process kinetics of the mentioned ions from single-component and four-component solutions was also studied by using the Weber–Morris model, the Boyd model and the method of moments. It was determined that the Weber–Morris model was the most accurately correlated with the experimental data [75].

In the case where the adsorption of the above-mentioned heavy metal ions with the initial concentration of 100 mg/dm^3^ was carried out in dynamic conditions, the ion extraction degree was arranged in the following series: Cu^2+^ (95.5%) > Pb^2+^ (89.6%) > Cd^2+^ (86.0%) > Zn^2+^ (60.0%) [76]. The experiments, performed in a process vessel with a pseudo-fluidized layer, confirmed that the mass-transfer process of Cd^2+^, Cu^2+^, Pb^2+^ and Zn^2+^ ions was adequately described with the homogeneous diffusion model in a solid phase [77].

The removal of Ni^2+^, Co^2+^, Cd^2+^, Cu^2+^, Pb^2+^, Cr^3+^ and Cr^6+^ ions from water solutions by their adsorption with activated coals, obtained by carbonization and subsequent activation with sulfuric acid (1:1) at 200 °C for 24 h, was studied. It was determined that the sorption capacity for heavy metal ions depends on the solution’s pH and was observed at pH = 1 for Cr^6+^ ions (34.7 mg/g) and at pH = 6 for the other metal ions, 33.57 mg/g for Cd^2+^ ions, 30.07 mg/g for Co^2+^ ions, 29.47 mg/g for Cr^3+^ ions, 27.21 mg/g for Ni^2+^ ions, 24.21 mg/g for Cu^2+^ ions and 22.85 mg/g for Pb^2+^ ions [78].

### 3.5. Extraction of Dyes from Aqueous Media by the Biomass of Apricot Kernel Shells

Activated carbons, obtained from agricultural raw material processing waste, were widely researched as adsorbents for removing various dyestuffs from model and sewage waters [79]. It was found that the adsorption characteristics of activated coals, obtained from various agricultural wastes, are different and heavily depend on the composition and structure of the raw material [80].

The adsorption process of Methylene blue dye with activated carbon, obtained from apricot kernel shells, was studied. It was determined that, at the initial dye concentration of 1.5 × 10^−5^ mol/dm^3^ and sorbent dosage of 33.3 g/dm^3^, the removal degree makes up over 99.25% [81]. It was found that the maximum sorption capacity, calculated from the Langmuir equation, was 4.11 mg/g and depended on the activation time. The adsorption isotherm was more accurately (R^2^ = 0.989) described with the Freundlich model [82].

The activated carbon, obtained from apricot kernel shells, was studied In the adsorption process of Astrazon Blue FGRL dye from model solutions with varying process parameters, such as the initial concentration of the dye (100–300 mg/dm^3^), adsorbent dosage (3–12 g/dm^3^) and temperature (303–323 K). The findings of the research demonstrated that the adsorption capacity of AC for the dye increased with the increase in the dye initial concentration, sorbent dosage and solution temperature. The adsorption isotherms are more accurately described with the Langmuir model, and the maximum sorption capacity, calculated from the Langmuir equation, amounts to 181.5 mg/g. The calculated thermodynamic characteristics demonstrated that this process is endothermic and spontaneous [83].

The activated coal, made of apricot kernel shells, was also studied for the adsorption of Coomassie Blue G-250 dye from model solutions. It was determined that the adsorption capacity for this dye was 10.09 mg/g at a temperature of 22.5 °C and 98.022 mg/g, at a temperature of 50 °C and pH ~ 2. It was identified that isotherms are the most accurately described with the Freundlich model, and the process kinetics is described with the pseudo-second-order model. Thermodynamic parameters (ΔG = −19.27 and −15.21 kJ/mol at temperatures of 295.5 and 329 °C, respectively, ΔH = −55.088 kJ/mol) indicated the process of chemisorption [84].

The process of Congo red dye adsorption with AC, obtained from apricot kernel shells, demonstrated that the maximum sorption capacity of the latter was 32.85 mg/g at 25 °C and pH = 13 and the initial dye concentration was 100 mg/dm^3^. The calculated thermodynamic parameters of the process indicate the spontaneous and endothermic nature of the adsorption process. It was determined that the adsorption isotherm as the most accurately described with the Dubinin–Radushkevich model, and the process kinetics with the pseudo-second-order model [85,86].

Three samples of AC were obtained from apricot kernel shells with various activation methods and were studied for removing the Safranine dye from model solutions. The following activation methods were used: vapor treatment, activation in nitrogen atmosphere, concentrated sulfuric acid treatment and mixed activation. It was found that the obtained activated carbons had a high sorption capacity for this dye of 243.9–294.1 mg/g [87].

The activated carbon, obtained by activating the apricot kernel shell carbonizates with sulfuric acid, was studied for removing the basic dye Astrazon yellow 7G with varying process parameters (dye concentration—50–300 mg/dm^3^, pH—4–10, sorbent dosage—2–8 g/dm^3^, temperature—25–50 °C and contact time of 35 min). It was determined that the highest sorption capacity value of 221.23 mg/g, calculated from the Langmuir equation, was observed at 50 °C and pH = 6. Adsorption isotherms were well described with the Langmuir and Freundlich models, depending on the solution temperature, and the process kinetics with the Weber–Morris model. Thermodynamic parameters indicated that the process was spontaneous and endothermic [88].

**Figure 11:**
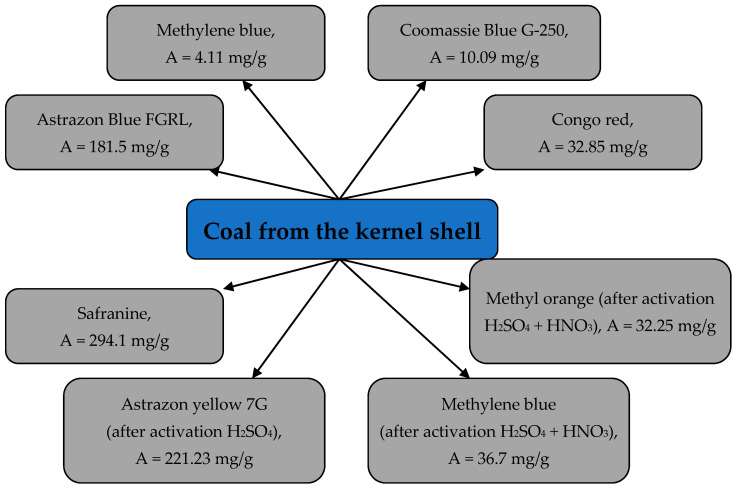
Dyes extracted from aqueous solutions with activated charcoal from apricot kernel shells.

The activated carbon, obtained by apricot kernel shell carbonization at 700 °C for 1 h and activated with a phosphoric–nitric acid mixture, was studied for the adsorption of Methylene blue and Methyl orange dyes with varying process parameters. It was found that the maximum sorption capacity for these dyes made up 36.7 and 32.25 mg/g, respectively. Adsorption isotherms are more accurately described with the Langmuir model, and the process kinetics with the pseudo-second-order model [89]. Examples of dyes extracted from aqueous solutions with activated charcoal from apricot kernel shells are shown in Figure 11.

### 3.6. Purification of Aqueous Media from Pharmaceutical Products and Pesticides

There are several publications, considering the usage of activated carbons, obtained from apricot kernel shells, for removing pesticides and pharmaceutical compositions from water media.

Three types of activated carbons, obtained from wood, walnut shells and apricot kernels, were studied for extracting the Atrazine pesticide and Cr^3+^ ions from model solutions individually or in the mixture with varying process parameters. It was found that the activated carbon made of apricot kernels shells had the smallest surface area among the used sorbents (276.15 m^2^/g). The values of the maximum sorption capacity of the AC for atrazine and chromium ions were found. For the activated carbons made of apricot kernels, these parameters made up 46.3 and 181.81 mg/g for the adsorption from individual pollutants’ solutions and 105.26 and 175.44 mg/g, respectively. The adsorption kinetics of atrazine and Cr^3+^ ions is described with the pseudo-second-order model. It was determined that the atrazine adsorption isotherms were more accurately described with the Freundlich model, and the Cr^3+^ ions adsorption isotherms with the Langmuir model [90].

The carbonizate, obtained from apricot kernel shells and activated with a H_3_PO_4_ solution, was used for removing tetracycline with varying process parameters. It was found that the specific surface area, total pore volume and average pore diameter of ACs made up 307.6 m^2^/g, 0.191 cm^3^/g and 1.957 nm, respectively. The maximum sorption capacity of the obtained activated carbon for tetracycline made up 308.33 mg/g. It was determined that the adsorption isotherm is more accurately described with the Freundlich model, and the process kinetics with the pseudo-second-order model [91].

### 3.7. Extraction of Petroleum and Its Products

Some more publications in the world literature are devoted to the extraction of petroleum and its processing products, as well as chemicals, resulting from organic synthesis, by using apricot kernel shell activated carbons [92].

The removal of petroleum and its products with activated coal, obtained from the carbonization of ground apricot kernel shells at 350 and 600 °C, was researched. It was determined that the higher carbonization temperature resulted in the formation of a larger surface area. It was also found that, with the decrease in the diameter of the AC particles, the sorption capacity for petroleum products increased. The maximum values of the sorption capacity of the activated carbons with a particle size 0.5 mm was determined, which made up 2.0 g/g for petroleum, 4.0 g/g for I-20A oil, 1.5 g/g for kerosene and 0.7 g/g for petrol [92].

The activated carbon, obtained by apricot kernel shell carbonization at 600 °C and, further, the mechanical activation with SAA with the subsequent additional activation in a CO_2_ flow, has the sorption capacity for diesel oil of 40 mg/g at an oil concentration in water of 200 mg/m^3^ [93,94].

The adsorption of phenol and nitrophenol with the activated carbon, obtained from apricot kernel shells, with varying process parameters, was studied. The activated carbon was obtained by activating the carbonizate with vapor at 700 °C. It was found that the AC surface area made up 1175 m^2^/g, and the total pore volume—0.93 cm^3^/g. It was determined that the maximum adsorption capacity for phenol, calculated from the Langmuir equation, made up 152 mg/g, and was 179 mg/g for nitrophenol [95,96].

The activated carbon, obtained by activating the apricot kernel shell carbonizates with phosphoric acid, was used for extracting phenol, meta-cresol and para-cresol, 2-chlorophenol, 4-nitrophenol, 2,4-dichlorophenol and 2,4-dinitrophenol. It was found that disubstituted phenolic derivatives were adsorbed in larger amounts than monosubstituted derivatives. It was determined that the maximum sorption capacities made up 101.7 to 533.25 mg/g [96,97,98,99,100,101,102,103,104,105,106,107].

Five types of activated carbons were made of various agricultural production wastes, including apricot kernel shells, and studied for extracting naphthalene from model solutions. It was found that the activated coal, obtained from apricot processing waste, had a low surface area and, consequently, the lowest value of maximum sorption capacity (175.44 mg/g) of the researched coal samples [97,98,99,100,101]. It was determined that all the adsorption isotherms were more accurately described with the Freundlich model, and the process kinetics with the pseudo-second-order model.

### 3.8. Brief Comparison of the Treatment Methods

The alkaline activation of apricot kernel shells with NaOH increased the maximum sorption capacity for Cu^2+^ ions by more than 4 times, from 2.5–4.83 mg/g to 10.8–12.25 mg/g. There was a slightly lower efficiency for the Pb^2+^ and Zn^2+^ ions, for which the increase in the sorption capacity occurred no more than 2 times: from 24.53 and 5.42 mg/g to 46.45 and 8.73 mg/g, respectively.

Other processing methods presented less efficiency. For example, the ultrasonic treatment of 200 W during 15 min with a frequency of 22 kHz helped to increase the maximum sorption capacity from 6.6 mg/g to 9.9 mg/g for Cr^6+^ ions.

Additionally, the most effective method is complex activation, in particular, the production of activated carbon obtained by carbonization and activation after treatment with sulfuric acid (1:1) at 200 °C for 24 h. In this case, the sorption capacity for Cr(VI) ions is 34.7 mg/g, 33.57 mg/g for Cd^2+^ ions, 30.07 mg/g for Co^2+^ ions, 29.47 mg/g for Cr^3+^ ions, 27.21 mg/g for Ni^2+^ ions, 24.21 mg/g for Cu^2+^ ions and 22.85 mg/g d for Pb^2+^ ions.

## 4. Conclusions

For the first time, the data concerning the usage of apricot (*Prunus armeniaca*) biomass components as sorption materials for removing various pollutants from water media were summarized. When processing hundreds of thousands of tons of apricots, thousands of tons of apricot kernels are generated as waste. It was established that the most studied components are apricot kernels shells. It was shown that effective sorption materials can be obtained based on apricot kernels for the removal of numerous pollutants from aquatic environments:

- Various ways of increasing the sorption capacity of apricot kernel biomass was analyzed. It was established that heat treatment at 250 °C is the most effective method.
The author’s microstructural studies proved that the specific structure of the biomaterial allows the retention of molecules and ions of pollutants. In this case, the heat treatment of the material leads to an increase in the defectiveness of the surface, increasing the sorption properties for many substances.
Ground apricot kernels were tested for the removal of heavy metal ions and dyes from the aquatic environment. Sonication or chemicals can improve the sorption characteristics of crushed apricot kernel shells. The sorption characteristics of ground apricot kernels shells can be increased by ultrasonic treatment. Thus, the ultrasonic treatment of 200 W during 15 min with a frequency of 22 kHz helps to increase the maximum sorption capacity from 6.6 mg/g to 9.9 mg/g for Cr^6+^ ions.
The influence of alkali treatment with an NaOH solution on the sorption capacity of apricot kernel shells for Cu^2+^, Pb^2+^ and Zn^2+^ ions was studied. It was determined that the alkali treatment increases the maximum sorption capacity from 4.83, 24.53 and 5.42 mg/g to 12.25, 46.45 and 8.73 mg/g, respectively.
The main way to use apricot kernels is the production of activated carbons. The choice of this material is explained by the fact that the shell of an apricot kernels is a low-ash material, and the high true density determines the possibility of obtaining solid adsorbents on its basis. In addition, this raw material already has a natural system of pores and channels in its structure, which can be developed through various carbonization and subsequent activation methods. Depending on their carbonization and activation parameters, activated carbons can have various surface areas ranging from 25 m^2^/g to 1200 m^2^/g. It is necessary to research the other components and waste products of apricot biomass processing for usage as sorption materials. The possibility of improving the sorption characteristics of apricot biomass with chemical or physical-chemical treatment was demonstrated.

As a direction for further research, it can be proposed to study the remaining components and waste from the processing of apricot tree biomass (leaves, bark and sawdust) as sorption materials. Sink for spent adsorbents or possible regeneration of adsorbents are also proposed for further studies. Another direction for further research is how pH affects the removal of heavy metals (cations vs. anions) and organic contaminants.

## Data Availability

Not applicable.
